# Predicting the Factors of Employee Agility Using Enterprise Social Media: The Moderating Role of Innovation Culture

**DOI:** 10.3389/fpsyg.2022.911427

**Published:** 2022-06-24

**Authors:** Luteng Zhang, Yan Xu, Chunchun Chen, Rui Zhao

**Affiliations:** ^1^College of Economics and Management, Shangqiu Normal University, Shangqiu, China; ^2^School of Transportation, Fujian University of Technology, Fuzhou, China; ^3^School of Management, Beijing Union University, Beijing, China

**Keywords:** agility, innovation culture, enterprise social media usage, communication quality, trust

## Abstract

This study aims to create a research model that examines how employee agility is affected by enterprise social media usage (ESMU), and to discuss the moderating role of innovation culture in communication quality, trust, and employee agility using the relational capital theory. Data of 477 Chinese employees from different companies were collected in this study for analysis, and the hypotheses developed were examined. The purpose of this study was to explore the influence mechanism that propels employees’ ESMU, communication quality and trust and the moderating effect of innovation culture. This study conducts PLS-SEM to analyze collected data. The results show that ESMU is positively associated with communication quality and trust; innovation culture plays a positive moderating role in ESMU and employee agility; and high communication quality and trust can lead to high agility. However, innovation culture does not have a remarkable moderating effect on ESMU and communication quality. This study offers empirical evidence on how the effect of ESMU on employee agility is transferred by innovation culture. In addition, the benefits of enterprise social media for organizational management are also measured in this study, which may motivate the managements to introduce enterprise social media in work spaces.

## Introduction

Common enterprise social media platforms including Wiki, Yammer, Slack, instant messaging, and microblogs allow enterprises to organize internal interaction, communication, and knowledge sharing on them (Leonardi and Meyer, 2015; Ding et al., 2019). Prior studies have confirmed benefits of enterprise social media usage (ESMU) for organizational work (Leonardi and Meyer, 2015; Pitafi et al., 2018b). For example, the favorable ESMU contributes to the enhancement of employee agility (Sherehiy and Karwowski, 2014; Cai et al., 2018; Pitafi et al., 2018c; Bala et al., 2019). Employee agility refers to the capability of employees to immediately and appropriately respond to external market exchanges (Sherehiy, 2008; Alavi et al., 2014; Alavi, 2016). Studies revealed that frequent communication and collaboration are requisite factors for the improvement of employee agility (Alavi, 2016; Bala et al., 2019). In particular, enterprise social media can help individuals to learn from colleagues in face of abrupt market changes (Leonardi, 2014; Pitafi et al., 2020), thus achieving favorable agility. However, very few studies of ESMU and employee agility were designed. As stated by scholars, ESMU can create a friendly environment where employees share and release market related information, interact with colleagues, and adapt to unforeseen circumstances (Wei et al., 2020). Other studies also present that ESMU can lower employees’ capability of perceiving and coping with market changes (Turban et al., 2011; Cao and Yu, 2019; Chen and Wei, 2019), because it will reduce the work efficiency and waste internet resources. These findings serve as a basis for further research on the usage of enterprise social media in work spaces.

One of the most challenging issue facing managers is how to run social media well in their organizations (Huy and Shipilov, 2012). Social media have been widely applied in many enterprises in China for work and social contact. Some scholars indicated that the usage of social media in enterprises can facilitate internal communication and social interaction, and can even enhance the job satisfaction and performance (Salehan et al., 2017; Zhang et al., 2019; Tajvidi and Karami, 2021). However, quite a few companies still do not allow employees to use social media during work, or fail to use social media as a supplement to their organizational operation. The reason may be that managers worry that social media usage at work could lead to procrastination, indulgence in pleasure, or misuse of time (Sun and Shang, 2014). But Huy and Shipilov (2012) found through comparative cases that organizations can benefit from the building of emotional capital by social media in the staff community. Therefore, building the relational capital among employees through social media has become an important and pressing issue.

Enterprise social media have a global influence on interaction, knowledge sharing, and communication (Pitafi et al., 2018c; Pivec and Maček, 2019) through changing the way employees communicate with each other, and offering platforms for information sharing (Pivec and Maček, 2019). Enterprise social media provide employees with a highly interactive and open communication platform (Ou and Davison, 2011), making communication among employees visible (Leonardi, 2014). For instance, information exchange on enterprise social media enables employees to easily review what they have said (Leonardi, 2014). ESMU encourages employees to express their own ideas freely, thereby facilitating their communication and collaboration (Riemer and Tavakoli, 2013; Treem and Leonardi, 2013). Furthermore, enterprise social media help to achieve the high-level communication quality and trust through transparent information, behaviors, preferences and knowledge exchange among employees (Leonardi and Meyer, 2015). On this basis, trust and communication quality provide additional opportunities for other employees to understand unexpected environmental changes (Treem and Leonardi, 2013; Cao and Ali, 2018), leading to the enhanced employee agility. Thus, this study attempts to explore how enterprise social media enhance employee agility through communication quality and trust based on the above research findings.

This study expects to (1) investigate employee agility through the effect of communication quality and trust in the context of enterprise social media; and (2) examine the moderating role of innovation culture on enterprise social media based the relational capital theory. This study has made contributions to existing theoretical literature from three dimensions. First of all, given that frequent communication and favorable information, as presented by prior studies, are potential factors for employee agility, this study examines the importance of employee agility from the perspectives of communication and information. Second, this study is conducted based on the renowned relational capital theory, which has been also applied in information system studies (Leonardi and Meyer, 2015; Chen and Wei, 2019). Third, the moderating effect of innovation culture is discussed in this study.

## Literature Review and Theoretical Background

### Relational Capital Theory

In the study of Kale et al. (2000) discussing the quality of relation between cooperative partners, relational capital theory was defined as the mutual trust, respect and friendship arising from close interaction between partners (Cummings, 2012). Generally speaking, relational capital is composed of three elements: trust, commitment, and communication (Sambasivan et al., 2011; Byun et al., 2018), but most scholars only concentrate on trust and communication (Collins and Hitt, 2006; Chen et al., 2016). Chen and Sharma (2013) found that communication quality and trust can have a significant effect on the use of social network sites. A further study conducted by Chen et al. (2016) presented that communication and trust can affect the sustained use of users for social network sites. In our context, social media play its part mainly through strengthening communication quality and trust in work and enhancing the employee agility. For these reasons, this study is designed based on the relational capital theory from two dimensions: communication quality and trust.

### Use of Enterprise Social Media in Work Spaces

Enterprise social media allows employees to obtain job-related information through communication, interaction, release, broadcasting, collaboration, and exchange in organizations (Leonardi and Meyer, 2015; Cai et al., 2018). Recently, many companies began to use enterprise social media tools as platforms for communication and collaboration among employees (DiMicco et al., 2008; Bala et al., 2019; Li et al., 2019). Composed by multiple communication tools, enterprise social media allow the information sharing, collaboration and access across time zones through more than one channels (Cao et al., 2012; Kanwal et al., 2018; Song et al., 2019). So far, a large number of organizations have used enterprise social media such as Microsoft’s Town Square and IBM’s Beehive for internally individual and job-related communication. Information in employee communication conducted on enterprise social media becomes transparent and visible, and may be available throughout the organization (Leonardi and Meyer, 2015; Leidner et al., 2018). Executives help employees achieve comparative advantages through a wide range of transparent information (Kane, 2015; Pitafi et al., 2020). Vuori (2012) discussed benefits of enterprise social media for enterprises, and contended that researchers should examine their usage in work spaces.

A majority of prior studies of ESMU were designed from a single perspective such as ESMU (Archer-Brown and Kietzmann, 2018; Bulgurcu et al., 2018; Leidner et al., 2018; Van Osch and Steinfield, 2018) and enterprise social software/website/platform usage (Kügler and Smolnik, 2013; Antonius et al., 2015; Kügler et al., 2015; Drummond et al., 2018). Specifically, related studies were conducted in terms of social media usage related to personal life (Nduhura and Prieler, 2017), social media usage related to society and job (Sun and Shang, 2014; Ma et al., 2020), social media usage related to job and entertainment (Huang et al., 2015), social media usage in and outside organizations (Forsgren and Byström, 2018), individual and organizational usage of social media (Fusi and Feeney, 2018), social media usage related to social contact, enjoyment and cognition (Ali-Hassan et al., 2015), and social media usage for consumption and contribution (Kuegler et al., 2015).

The usage of social media in organizations has been verified in most previous studies (van Zoonen and Rice, 2017; Tajudeen et al., 2018; Zha et al., 2018), but researchers seldom discuss the effect of ESMU on employee agility. Therefore, it is of great concern to understand ESMU (Odoom et al., 2017; Schlagwein and Hu, 2017).

### Effect of Enterprise Social Media Use on Communication Quality

Communication quality is defined as timely, accurate and abundant information sharing (Spralls et al., 2011). Communication quality is an individual capability that can be increased through frequent communication with other individuals (Yin et al., 2018). Enterprise social media motivate employees to release contents online for the development of high communication quality (Leonardi et al., 2013). Enterprise social media has been widely used in different organizations with the booming social media in recent years (Alimam et al., 2017; Cai et al., 2018). The extensive use of social media in organizations boost the sharing of information and knowledge among employees (Archer-Brown and Kietzmann, 2018; Garcia-Morales et al., 2018). Decision makers believe that high-quality communication is based on the frequency of information flow (Orpen, 1997). Research shows that social media has positive effects on communication, cooperation, knowledge sharing (Leonardi, 2014), and communication quality (Korzynski, 2014). The usage of social media in organizations can definitely improve the communication efficiency of employees (Korzynski, 2014). The appropriate use of enterprise social media can narrow the gap between employees, and improve the communication quality (Ou and Davison, 2011; Pitafi et al., 2018b).

Gibbs et al. (2015) found that ESMU has boosted the cross-border communication quality of a large-scale telecommunications company in Russia. Ou et al. (2014) reported that the usage of enterprise social media in work spaces has a positive effect on communication quality. As a result, we contend that the communication timeliness, accuracy, and abundance among employees will be enhanced if enterprise social media are used in organizations for discussion about work and arrangement of social activities between colleagues, thus improving the communication accessibility and efficiency and raising the communication quality. Based on the above statements, a hypothesis is developed as follows in this study:

H1: ESMU is positively associated with communication quality.

### Effect of Enterprise Social Media Usage on Trust

Trust is defined as an expectation that the other party can act and perform his or her obligations in a predictable manner, and deal with affairs impartially (Chen and Sharma, 2013). Trust has been considered as an important factor that affect employee outcomes such as satisfaction and performance (Top et al., 2015; Li et al., 2018). Studies demonstrate that trust is a critical factor for business success and human resource practices (Morgan and Zeffane, 2003).

With regard to the relationship between ESMU and trust among employees, prior studies revealed that the specific way of individual using technologies will affect the trust among employees (Valenzuela et al., 2009). Zhao and Rosson (2009) investigated the relationship between contents on enterprise social media and trust, finding that ESMU has a significant effect on trust. In effect, enterprise social media can affect the social trust when they are used for work and social contact (Kobayashi et al., 2006; Räsänen and Kouvo, 2007; Valenzuela et al., 2009). The use of social media platforms for the same purpose can improve the trust, because a work group can be established on such platforms (Yuan and Liu, 2017). Employees who use these applications gradually build relationship capital and trust among them as communication increases (Sun and Shang, 2014; Cheng et al., 2017). The increasingly frequent communication can gradually build trust among employees using these Apps (Sun and Shang, 2014; Cheng et al., 2017). Some scholars also pointed out that ESMU can strengthen the trust among colleagues (Gil de Zúñiga et al., 2012). In this context, we also contend that ESMU for work and social contact can positively affect the trust among employees. On this basis, we propose a hypothesis as follows:

H2: ESMU is positively associated with trust.

### Communication Quality, Trust, and Employee Agility

Denning (2017) highlighted in his study of strategic management that agility is a driving force of business development. Agility can also accelerate innovations in products, services and business models for business growth (Bouncken et al., 2021). In this case, agility has become an important factor that has a growing influence on organizational performance (Aravind Raj et al., 2013).

It is commonly accepted that employee agility is based on their efficient and effective response to market changes (Alavi et al., 2014; Alavi, 2016; Pitafi et al., 2020). The effectiveness of responses reflects the communication quality. The communication quality achieved when enterprise social media are used in work can lower the work complexity and facilitate to solve task-related and interpersonal issues, contributing to the agility enhancement (Zhang et al., 2016). Employees can exchange ideas and information through enterprise social media, which further leads to higher job performance and increased work efficiency and agility (Neeley and Leonardi, 2018). Recent research has also examined the relationship between communication and work outcomes (Hsu et al., 2012). Prior studies presented that the increased use of enterprise social media in work spaces may enhance the social interaction and communication quality (Markman, 2015). The resulting high communication quality, mutual understanding and trust may allow enterprises to train employees that are willing to share more market knowledge with colleagues (Chen and Wei, 2019), thus contributing to the enhancement of work agility. Thus, a reasonable assumption is that employees will react to and adopt market changes more appropriately when higher communication quality leads to timely, accurate and relevant sharing of information *via* ESM usage (Ou and Davison, 2011). Thus, we can see that this study is supported by literature. A hypothesis is developed as follows:

H3: Communication quality is positively related to employee agility.

Gil de Zúñiga et al. (2012) examined the trust relationship between enterprises and social media. Studies do presented that information sharing and cooperation will be more facilitated when partners trust in each other (Butler, 1999). As defined in these studies, cooperation and its perquisites are expected to become important sources of labor agility (Storme et al., 2020).

Trust was considered to be positively associated with agility performance in prior studies. Trust can motivate active engagement of employees, and encourages them to explore and grasp new opportunities actively and expeditiously (Kark and Carmeli, 2009). In addition, with the trust, employees are more content to pursue their career goals and adapt to ever-changing situations (Edmondson, 1999; Edmondson et al., 2001). This kind of trust can also facilitate the agility enhancement through impelling employees to express their own opinions (Edmondson, 1999; Detert and Burris, 2007). Thus, trust is the basis for employees to generate agile behaviors (Nold and Michel, 2016), and allows employees to seize opportunities, adapt to changes and develop an independent mind, thereby optimizing their agility performance. As argued by Ou and Davison (2011), communication quality is able to enhance the personal agility in work. Tijunaitis et al. (2019) found that respondents using social media in work reported higher agility than those not using social media to communicate with colleagues.

H4: Trust is positively related to employee agility.

### Moderating Role of Innovation Culture

It is demonstrated in literature that enterprise social media tools have inconsistent effects on employee agility (Ali-Hassan et al., 2015; Cao et al., 2016), which gives us a chance to investigate the causes (e.g., innovation culture as a moderator) for such inconsistencies.

Dobni (2008) defined innovation culture as the innovative spirit and materials developed by enterprises in the innovation process and management activities, including innovative values, codes of conduct, systems and norms, material cultural climate, and other innovation-related elements. In fact, the key to innovation can be described as creativity, openness, receptivity to new ideas, risk-taking spirit, and entrepreneurial mindset from the angle of culture. Thus, companies advocating innovation often motivate employees to think, strengthen communication and information exchange, and accept new ideas (Ali-Hassan et al., 2015). Furthermore, if organizations can form an atmosphere of cultural innovation that is perceived as positive by individuals, it is more likely to achieve smooth communication (Shanker et al., 2017), and trust also takes shape in this process (Ruppel and Harrington, 2000). Agility generated in this process, directly or indirectly, depends on whether the organizational culture is an innovative one. We contend that agility is easier to produce under an innovative environment. Based on the above, we propose the following hypotheses:

H5a: Innovation culture moderates relationship between enterprise social media usage and communication quality.
H5b: Innovation culture moderates relationship between enterprise social media usage and trust.

**FIGURE 1:**
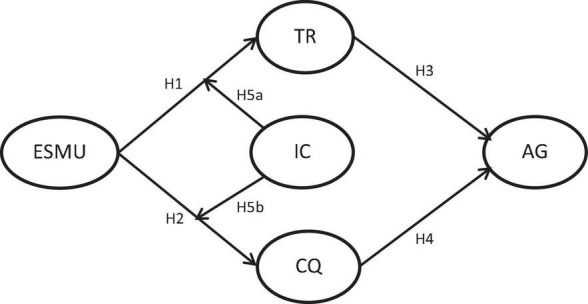
Research framework.

According to the above hypotheses, the research framework is shown in Figure 1.

## Data Collection

A questionnaire was designed for this study to collect data related to user demographics and relevant characteristics of enterprises. In order to enhance the sample representativeness, researchers select effective sample clusters based on their research purposes and issues. Thus, purposive sampling is adopted, and several conditions will be established during sampling so as to improve the representativeness of the research samples. In the period from August to October 2021, we distributed questionnaires to 600 employees using enterprise social media in work spaces. Later, we made follow-up calls and sent reminder e-mails to participants, and offered USD 2 as a reward for their response. Managers in involved companies also offered us a hand, which greatly enhanced the response rate. We withdrew 572 questionnaires within 6 weeks, including 95 invalid ones because of information omission or improper completion. In a word, we obtained 477 questionnaires with complete answers, resulting in a response rate of about 83.4%. We analyzed the potential non-response bias by comparing the initial 25% and the final 25% of all structural samples. The results demonstrate that the differences of all structural means were not statistically significant, which verified the indifference of non-response rate in this study. Table 1 shows the demographic data of the samples.

**TABLE 1:** Analysis of sample demographic variables (*n* = 477).

Measure	Item	N (Percentage %)
Gender	Male	247 (51.7%)
	Female	230 (48.3%)
Age	21–30	193 (40.5%)
	31–40	163 (34.2%)
	41–50	72 (15.1%)
	Over 50	49 (10.2%)
Education	Senior high school or below	29 (6.0%)
	College	338 (70.9%)
	Graduate school or above	110 (23.1%)
Monthly income	Less than CNY 2000	117 (24.6%)
	Between CNY 2001 and less than CNY 5000	151 (31.7%)
	Between CNY 5001 and less than CNY 8000	154 (32.2%)
	Greater than CNY 8001	55 (11.5%)
	Total	477 (100%)

Questionnaires used in this study were prepared according to generally accepted practices (Cao et al., 2020), and all the items were extracted from literature. We also invited one professor, three associate professors and one postgraduate to review the designed questionnaire, as a means to correcting any content that is inconsistent and difficult to comprehend. When self-report questionnaires are used to collect data at the same time from the same participants, common method variance (CMV) may be a concern. A *post-hoc* Harman one-factor analysis was used to test for common method variance (Podsakoff and Organ, 1986). The explained variance in one factor is 40.554%, which is smaller than the recommended threshold of 50%. Therefore, CMV is not problematic in this study to test the research model, a survey instrument was developed with each construct measured using multiple items (Harman, 1976). Most items were adapted from existing measures in the related literature with confirmed content validity and reliability, and then modified to fit our research context. Enterprise Social Media Usage was measured by four items adapted from Ou and Davison (2011). Communication Quality were measured by four items adapted from Ou and Davison (2011). Trust were measured by four items adapted from Spralls et al. (2011). Agility was measured by three items adapted from Alavi et al. (2014). Innovation Culture was measured using Wallach (1983) instrument (four items). All items were measured with a five-point Likert scale (1 = totally disagree; 5 = totally agree).

### Evaluation of Measurement Model

Data analysis was divided into two stages: the reliability and validity of the measurement model were evaluated in the first stage, and the structural model was examined in the second stage to conduct the examination of the research hypotheses (Hair et al., 2013). In this study, the latent variable structural equation models (SMEs) of SmartPLS3.0 and SPSS 25 were adopted as the analysis method. Table 2 shows the average number, factor loading, reliability, and average variance extracted (AVE) value of each construct in this study. The composite reliability (CR) of each construct in this study ranged from 0.911 to 0.951, and every Cronbach’s alpha (α) was higher than 0.7, indicating a high reliability for the constructs in this study. AVE ranged from 0.720 to 0.830, which was also greater than 0.500, indicating a good convergent validity for the constructs in this study. Table 3 shows the correlation coefficient matrix for each construct in this study. The square root of the AVE for each construct was greater than the correlation coefficient for the dimensions (Chin, 1998), indicating a good discriminant validity for the constructs in this study. Besides, Henseler et al. (2016) have proposed that the heterotrait-monotrait ratio (HTMT) of correlations based on the multitrait-multimethod matrix could be adopted as a method to determine discriminant validity. Table 4 shows that the HTMT values for the constructs are all lower than 0.9, showing a good discriminant validity for the constructs in this study. The above analysis showed a good construct validity for this study.

**TABLE 2:** Summary of study measures, factor loadings of CFA and SEM.

Constructs	Standardized loadings	α	CR	AVE
Enterprise Social Media Usage (ESMU)		0.877	0.915	0.730
ESMU 1	0.852			
ESMU 2	0.879			
ESMU 3	0.853			
ESMU 4	0.853			
Communication Quality (CQ)		0.870	0.911	0.720
CO 1	0.838			
CO 2	0.856			
CO 3	0.882			
CO 4	0.816			
Trust (TR)		0.903	0.932	0.775
TR 1	0.892			
TR 2	0.883			
TR 3	0.860			
TR 4	0.887			
Agility (AG)		0.892	0.925	0.755
AG 1	0.843			
AG 2	0.906			
AG 3	0.864			
AG 4	0.861			
Innovation Culture (IC)		0.932	0.951	0.830
IC 1	0.899			
IC 2	0.922			
IC 3	0.918			
IC 4	0.905			

*SD, Standard deviation; CR, Composite Reliability; AVE, Average Variance Explained.*

**TABLE 3:** Matrix of construct correlation coefficients.

Constructs	1	2	3	4	5
1. ESMU	0.854				
2. TR	0.531	0.88			
3. AG	0.540	0.447	0.869		
4. IC	0.565	0.465	0.497	0.911	
5. CQ	0.577	0.531	0.574	0.528	0.849

*The diagonal line is the square root of the average variance extracted (AVE) of each dimension.*

**TABLE 4:** Heterotesait-monotrait (HTMT).

Constructs	1	2	3	4	5
1. ESMU					
2. TR	0.593				
3. AG	0.608	0.495			
4. IC	0.626	0.504	0.544		
5. CQ	0.658	0.598	0.647	0.582	

### Data Analysis and Results

Smart PLS 3.0 was adopted for the structural model analysis in this study. The value of the standardized root mean square residual (SRMR) can be applied to evaluate the fit of the research model, which is between 0 and 1: the closer it is to 0, the better the fit is. However, the saturated model of the SRMR assumes that the number of paths in the structural model is the same as the number of related constructs in the measurement model, and the estimated model is calculated in terms of the sample dataset itself and the rows. When the SRMR of the saturated model and the estimated model is less than 0.08, it indicates a good fit for the model (Hu and Bentler, 1999). The value of the normed fit index (NFI) is between 0 and 1, where the closer it is to 1, the better the fit is, and a value of NFI greater than 0.8 means an acceptable fit.

According to the results calculated by Smart PLS, the value of the SRMR for the saturated model in this study is 0.045, and the value of the SRMR for the estimated model is 0.076, both of which are less than 0.080. The value of NFI is 0.894, which meets the requirements for fit. There is thus a good model fit for this study.

**FIGURE 2:**
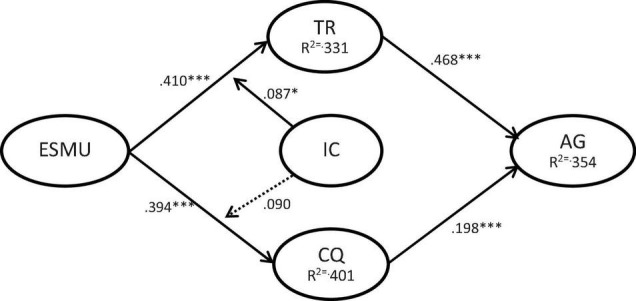
SEM analysis of the research model. *p < 0.05; ***p < 0.001.

After the evaluation and measurement results were found to be satisfactory, we evaluated the structural model, and examined the hypothesis through the percentage of variance and the significance of structural path. Figure 2 shows the test results of the PLS analysis containing control variables. Enterprise social media usage was positively correlated with trust (β = 0.410, *p* < 0.001) and communication quality (β = 0.394, *p* < 0.001), thus supporting H1 and H2. Trust (β = 0.468, *p* < 0.001) and communication quality (β = 0.198, *p* < 0.001) had a significant positive correlation with agility, thus supporting H3 and H4. Our findings also evidence that innovation culture significantly moderates the relationships of enterprise social media usage to trust (β = 0.087, *p* < 0.05), thus, H5a is supported. But culture not significant moderates the relationships of enterprise social media usage to communication quality (β = 0.090, *p* = 0.057), thus, H5b is not supported.

**FIGURE 3:**
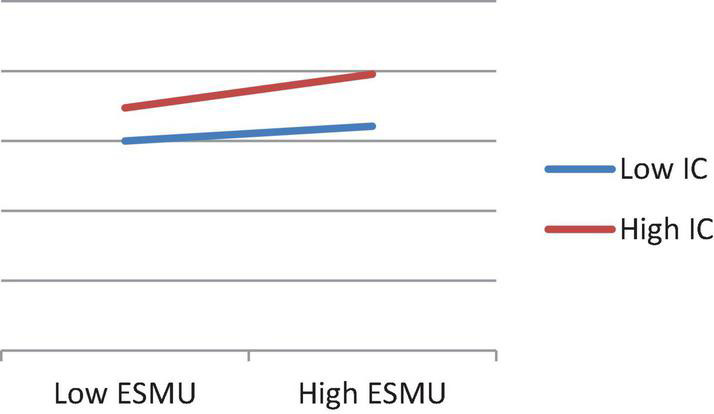
Interaction effects.

To show the moderating effects among these relationships clearer, we plotted these significant interactions and indicated that as shown in the simple slope chart in Figure 3.

Innovation culture has a remarkable moderating effect on ESMU and trust. To show the moderating effects among these relationships clearer, we plotted these significant interactions, and indicated that ESMU is more capable of predicting trust more significantly under a high level of innovation culture, as shown in the simple slope chart in Figure 3.

## Discussion

This study aims to discuss how ESMU can improve the employee agility. We considered the work and social purposes of social media, and examined the moderating effect of innovation culture on the relationship between social media and employee agility. Interestingly, the favorable use of enterprise social media is conducive to higher communication quality and enhanced trust among employees. More importantly, it is found that innovation culture can moderate the relationship between ESMU and communication quality.

The contributions made by this study can be reflected from the following three aspects. First of all, this study examines the effect of ESMU exerted through communication quality and trust on employee agility. Employee agility is a critical research subject that is to be explored. Current data sets support a majority of hypotheses, including the moderating effect. As stated in H1 and H2, ESMU is positively associated with communication quality and trust. We draw conclusions that agree with previous studies (Ou and Davison, 2011). Ou et al. (2014) also presented that the application of instant messaging in work spaces can enhance the communication quality. Chen and Wei (2019) verified the significant correlation of ESMU with trust.

Second, as can be seen from our research findings, communication quality and trust have a significant impact on employee agility, and play a moderating role in the relationship between ESMU and employee agility, so H3 and H4 are supported. The high communication quality enables employees to timely and properly deal with abrupt market-related changes, because ESMU makes other communications related to market changes simpler and more effective. This result is consistent with evidence provided in previous studies of social media environment and job performance (Cao et al., 2012; Moqbel et al., 2013; Pitafi et al., 2018a). Moreover, employees can obtain knowledge in time from enterprise social media (Treem and Leonardi, 2013), which will promote knowledge sharing and information exchange with other network members. All these findings prove that high communication quality can guarantee employees to find work-related resources in time, thus contributing to the enhancement of their work agility.

Third, this study investigates the moderating effect of innovation culture on the relationship of ESMU with communication quality and trust. Our results demonstrate that innovation culture has a positive moderating effect on the relationship between ESMU and trust, which agrees with previous research results of other scholars (Valenzuela et al., 2009; Gil de Zúñiga et al., 2012), but does not moderate the relationship between ESMU and communication quality. Thus, the current data does not support H5b. Enterprise social media are open platforms, on which companies advocating innovation culture encourage employees to release, broadcast and exchange information with others. Other irrelevant employees may also receive the same contents of communication, which are redundant information for them and may create additional pressure and have an adverse effect on communication quality (Cao and Yu, 2019; Chen and Wei, 2019). According to our speculation, employees in organizations advocating innovation culture may think that using enterprise social media in work spaces can cause waste of time and energy, and further lead to reduction in job performance.

### Theoretical Implications

Findings of this study provide some theoretical contributions to literature of enterprise social media and employee agility. First, our findings validate the positive effect of communication quality and trust on employee agility. Prior studies revealed that socialization and psychological status are the key to the relationship between ESMU and job performance (Cao et al., 2012; Moqbel et al., 2013; Cai et al., 2018). However, there is no research that verifies the moderating effect of innovation culture on the relationship between ESMU and employee agility.

Second, existing studies discussing the moderating role of enterprise innovation culture also revealed that innovation culture can intensify the relationship between ESMU and trust. Our findings, however, show that innovation culture does not strengthen the relationship between EMSU and communication quality. Due to the openness of enterprise social media, all posts or broadcast messages on it are also available for other relevant individuals (Leonardi, 2014; Zha et al., 2018). A mass of communication contents will bring pressure for employees (Cao and Yu, 2019), and lower their job performance. This is caused by the unbalanced use of enterprise social media technology.

Third, this study has driven the development of the relational capital theory. Focusing on the relational capital theory (Treem and Leonardi, 2013; Ellison et al., 2015), previous studies discussed relational commitment (Chen et al., 2013), loyalty (Cheng et al., 2017), learning (Kale et al., 2000), value creation (Sambasivan et al., 2011), productivity (Kweh et al., 2014), innovation (Vlaisavljevic et al., 2016), competitive advantages (Wang, 2014), continued use of websites (Chen et al., 2016), and final relational performance (Kohtamäki et al., 2012, 2013). However, this study uses the relational capital theory to examine ESMU at the practical level, which is a pioneer contribution to the theory.

### Practical Implications

This study provides important guidelines for the enhancement of employee agility. First, the positive characteristics of ESMU such as high communication quality and trust are highlighted in this study. Previous studies have reported the negative influence caused by the usage of enterprise social media in work spaces (Cao and Yu, 2019; Chen and Wei, 2019). Chen and Wei (2019) concluded that the characteristic of enterprise social media will lead to the social- and work-related information overload. Under an innovation culture context, when considering how to improve employee trust, ESMU can be used for work-related purposes. One possible reason may be that when QQ is used for work-related purposes, the innovation culture encourages employee communication and sharing, which contributes to employee social capital (Sun and Shang, 2014) and trust (Cheng et al., 2017). By contrast, we offer new insights to managers and human resource management, and help employees using enterprise social media in work spaces enhance their agility. As indicated in this study, ESMU is positively associated with communication quality and trust. Most organizations do not use enterprise social media, because they worry about waste of time and resources and reduction of personal job performance due to chatting on them (Cao and Yu, 2019). Therefore, the results of this study persuade executives to believe that communication *via* enterprise social media is beneficial.

Second, it is reported in this study that the development of employee ability is a complex process (Cai et al., 2018; Pitafi et al., 2018c; Bala et al., 2019), and that high communication quality and trust are positively correlated with employee agility. For this reason, we suggest that enterprises should make good use of this technology to increase the efficiency of enterprise management, enhance employee agility and motivate employees to face changes and risks in work through, for example, organizing discussion groups, seminars, and knowledge sharing events on enterprise social media.

Third, we find in this study that innovation does not moderate the relationship between ESMU and communication quality. On this account, we recommend to design some filtering applications to keep the focus of employees on useful contents, or to provide some functions to allow communication restrictions at certain times to maintain the degree of concentration. We believe that this study will guide managers to understand the benefits of ESMU, and promote enterprises using enterprise social media in work spaces to do a good job in management.

### Limitations and Future Research Directions

Nevertheless, limitations can still be found in this study. First, like all empirical studies, there are some limitations to our study. Firstly, empirical studies are limited by the intrinsic methodology. This study is limited to cross-sectional empirical research. A longitudinal database could be established in subsequent studies to observe how the effect of ESMU on employee agility is transferred by innovation culture. Second, the use intensity of enterprise social media may cause variations to the research results, so researchers can further verify existing results by taking the use intensity into account. Besides, in this study, despite there is no difference in all the variables about income, previous studies have indicated that different income and employee’ genders result in different agility, thus it is necessary to incorporate income and genders into issues related to EMSU. As the study aims to strengthen the degree of theoretical generalization, the influence that gender and income bring to the research model is not discussed in the research findings. As a consequence, it is suggested that subsequent researchers can add the variable of employee’ background for comparative analysis to provide more valuable insights and enrich theoretical connotations.

## Data Availability Statement

The original contributions presented in the study are included in the article/supplementary material, further inquiries can be directed to the corresponding author.

## Ethics Statement

The studies involving human participants were reviewed and approved by Ethics Committee of Beijing Union University. The patients/participants provided their written informed consent to participate in this study.

## Author Contributions

LZ, YX, and CC contributed to the ideas of educational research, collection of data, and empirical analysis. LZ and RZ contributed to the data analysis, design of research methods, and tables. LZ participated in developing a research design, writing, and interpreting the analysis. All authors contributed to the literature review and conclusions and approved the submitted version.

## Conflict of Interest

The authors declare that the research was conducted in the absence of any commercial or financial relationships that could be construed as a potential conflict of interest.

## Publisher’s Note

All claims expressed in this article are solely those of the authors and do not necessarily represent those of their affiliated organizations, or those of the publisher, the editors and the reviewers. Any product that may be evaluated in this article, or claim that may be made by its manufacturer, is not guaranteed or endorsed by the publisher.
